# Metabolomics Reveals the Dynamic Characteristics of Flavonoids During the Different Developmental Stages of *Citrus reticulata* ‘Shiyue Ju’ and Its Mutant *C. reticulata* ‘Denglong Ju’

**DOI:** 10.3390/cimb48080774

**Published:** 2026-07-29

**Authors:** Qin Guan, Doudou Huang, Lan Zhang, Zongyou Lv

**Affiliations:** 1 Guangxi Key Laboratory of Fruits and Vegetables Storage-Processing Technology, Guangxi Academy of Agricultural Sciences, Nanning 530007, China; gqsxn047722@126.com; 2 The SATCM Key Laboratory for New Resources & Quality Evaluation of Chinese Medicine, Institute of Chinese Materia Medica, Shanghai University of Traditional Chinese Medicine, Shanghai 201203, China; hdd890920@163.com

**Keywords:** metabolomics, flavonoids, citrus, dynamic changes, polymethoxylated flavones

## Abstract

Flavonoids, prominent bioactive compounds in citrus fruits, exhibit diverse health-promoting properties. However, the dynamic compositional changes in flavonoids during fruit development remain insufficiently characterized in *Citrus reticulata* ‘Shiyue Ju’ (STJ) and *C. reticulata* ‘Denglong Ju’ (DLJ). In this study, a comprehensive untargeted metabolomics approach was employed to systematically analyze the flavonoid profiles in both peel and flesh tissues across six key developmental stages, leading to the putative annotation of 205 flavonoids. Heatmap analysis revealed higher flavonoid levels in SP-4 (STJ peel in stage 4) compared to DP-4 (DLJ peel in stage 4), potentially resulting from the substantial upregulation of genes involved in flavonoid biosynthesis during this period. Furthermore, S-plot analysis identified salvigenin, demethylnobiletin, and nobiletin as key discriminators in the peel of STJ (SP), whereas sinensetin, tangeretin, and 3′,4′,5,7-tetramethoxyflavone were predominant in the peel of DLJ (DP). These compounds could represent potential biochemical markers for varietal differentiation. Notably, SP exhibited higher levels of neohesperidin, hesperidin, naringin, and naringenin, suggesting enhanced health-promoting properties. The results enhance our understanding of flavonoid dynamics during citrus development and provide valuable implications for quality-oriented cultivation and genetic improvement.

## 1. Introduction

As an important economic crop, citrus is extensively cultivated in tropical and subtropical regions worldwide, including China, Brazil, India, and Mexico [1]. Citrus fruits are rich in various bioactive compounds, such as dietary fiber, essential oils, carotenoids, and notably flavonoids, which demonstrate significant biological activities including antioxidant, anti-inflammatory, and anticancer properties [2,3]. These compounds are particularly concentrated in peel tissues. Structurally, flavonoids are characterized by a C6-C3-C6 flavone backbone, typically substituted with hydroxyl, methoxyl, or glycosyl groups [4]. To date, more than 80 flavonoids have been identified in *Citrus* species, which are classified into six major subclasses: flavones, flavanones, flavonols, dihydroflavonols, isoflavanones, and anthocyanins [5]. Their diverse chemical structures contribute to a broad spectrum of health benefits and functional applications in food, pharmaceutical, and cosmetic industries [6].

*Citrus* species have undergone substantial varietal differentiation driven by interspecific hybridization and anthropogenic management [7]. In China, *Citrus reticulata* ‘Shiyue Ju’ (STJ) and its derived bud mutant, *C. reticulata* ‘Denglong Ju’’ (DLJ), are commercially significant cultivars extensively cultivated in Guangdong and Guangxi provinces [8,9,10]. These two cultivars display pronounced phenotypic divergence in fruit traits, particularly in shape and coloration, which is paralleled by preliminary metabolomic evidence [11]. Although a previous study characterized the mature-stage metabolomic profiles of STJ and DLJ at a single mature stage [11], the temporal dynamics of flavonoid accumulation during fruit development remains unexplored. To address this gap, the present study provides the comprehensive characterization of the temporal accumulation patterns of major flavonoid classes, including flavanones, flavonoid glycosides, and polymethoxyflavones, across six distinct developmental stages, from young fruit to full maturity, in both cultivars. Therefore, elucidating the temporal variations in flavonoid profiles between STJ and DLJ throughout maturation will provide novel insights into the metabolic mechanisms underlying quality formation in citrus mutants, thereby facilitating the identification of precise targets for marker-assisted breeding.

Metabolomics, a rapid and reproducible analytical approach, has been extensively utilized to investigate dynamic intracellular metabolic adaptations in plants [12,13]. Meanwhile, multivariate statistical techniques, including principal component analysis (PCA) and orthogonal partial least squares-discriminant analysis (OPLS-DA), have proven highly effective in elucidating the contributions of specific metabolites to critical quality attributes, including flavor profiles and bioactive compound composition [14,15]. Therefore, the integration of metabolomics with these multivariate methods provides a robust strategy for systematically characterizing differential metabolites during dynamic developmental processes, as demonstrated in previous studies [16,17].

In this study, comparative metabolomic analysis was conducted to elucidate varietal differences in flavonoid composition between STJ and DLJ. Using a metabolomics approach, dynamic changes in the flavonoid profiles of both varieties were tracked across six developmental stages. Multivariate statistical and visualization techniques, including heatmap, PCA and OPLS-DA, were applied to delineate stage-specific flavonoid metabolic characteristics. Comparative analysis further identified differentially accumulated flavonoids, highlighting the key compounds during fruit maturation. This study provides a systematic comparison of dynamics in flavonoid metabolites during the developmental stages of STJ and DLJ, contributing to both theoretical knowledge and practical applications in citrus variety improvement.

## 2. Materials and Methods

### 2.1. Plant Materials

**Figure 1:**
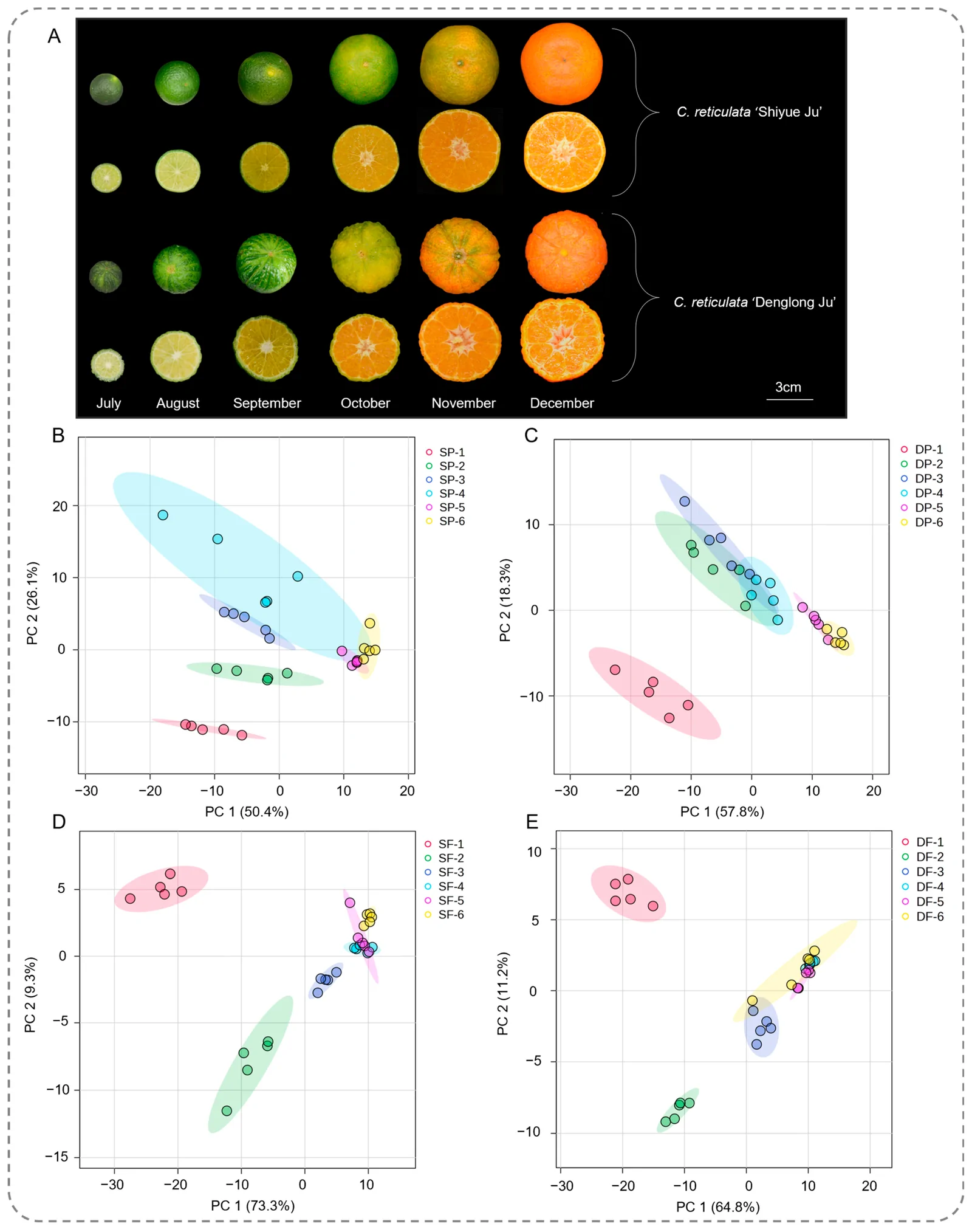
(**A**) The phenotypes of fruits in STJ and DLJ at six changing stages; PCA score map of SP (**B**), DP (**C**), SF (**D**), and DF (**E**) with different growth stages.

A total of 120 fruit samples of STJ and DLJ (2 cultivars × 2 tissues × 6 developmental stages × 5 biological replicates, detailed in Supplementary Table S1) were collected during an independent growing season (2024) from the Guangxi Academy of Agricultural Sciences in China (22.8410 °N, 108.2497 °E). The plants were cultivated under uniform agronomic conditions with natural light, drip irrigation, fertilization and routine pest management. To ensure that temporal variations reflect genuine developmental trajectories, individual trees were permanently labeled at the onset of the experiment. As illustrated in Figure 1A, the same set of tagged trees (*n* = 5 per cultivar) was sampled longitudinally across all six developmental stages (July through December). Each biological replicate was constituted by pooling peel and flesh tissues from the tagged trees of the same cultivar at an equivalent developmental stage. From each tree, 15–20 uniformly sized, disease-free fruits were harvested. Following collection, the peel and flesh tissues were separated, immediately flash-frozen in liquid nitrogen, and stored at −80 °C until metabolite profiling.

### 2.2. Flavonoid Extraction and UHPLC-Q-TOF-MS Conditions

Flavonoid compounds were extracted from the STJ and DLJ samples following the method described by Guan et al. [18]. Briefly, freeze-dried samples (50 mg) were extracted with 2 mL of 70% methanol at 4 °C for 12 h and sonicated at 25 °C for 30 min. After centrifugation at 13,000 rpm for 15 min, the supernatants were filtered through a 0.22 µm membrane prior to UHPLC-Q-TOF-MS analysis. To ensure system stability, pooled quality control (QC) samples were injected after the initial four analytical runs and subsequently after every ten samples.

Warfarin (0.2 μg/mL) was selected as the internal standard due to its physicochemical similarity to flavonoids in terms of polarity and solubility, as well as its natural absence in citrus tissues, which eliminates endogenous interference. Additionally, warfarin exhibited a distinct RT and maintained robust stability throughout the LC-MS/MS analytical workflow. The prepared samples were determined by an Agilent 1290 UHPLC system (Agilent Technologies, Santa Clara, CA, USA) equipped with an ACQUITY UPLC BEH C18 column (2.1 × 100 mm, 1.7 µm; Waters, Milford, MA, USA). The mobile phase consisted of (A) 0.1% (*v*/*v*) aqueous formic acid and (B) acetonitrile. Sample measurements were performed with the following gradient program: 0–10 min, 5–30% B; 10–12 min, 30–45% B; 12–22 min, 45–95% B; 22–30 min, 95% B. Operational parameters included a flow rate of 0.3 mL/min, a column temperature of 50 °C, and an injection volume of 2 μL. All samples were maintained at 4 °C to ensure analyte stability during analytical sequences.

The TOF MS spectrometer (6530B, Agilent Technologies) was operated and followed by the ESI ion source parameters: heater temperature: 350 °C; capillary voltage (IS): 4000 V (positive ion mode); nebulizing gas pressure was set at 45 psi; gas flow: 8.0 L/min; and declustering potential: 60 V. For data collection, the RT was in the range 0–30 min and mass in the range 100–1000 Da.

### 2.3. Qualitative Analyses of Metabolites

Flavonoid identification was primarily based on accurate mass (m/z) matching (±5 ppm) against in-standards and online databases (e.g., METLIN (http://metlin.scripps.edu/index.php, accessed on 20 April 2025), MassBank (http://www.massbank.jp/, accessed on 20 April 2025), PubChem (http://pubchem.Ncbi.nlm.nih.gov, accessed on 20 April 2025), HMDB (http://www.hmdb.ca/, accessed on 20 April 2025), and NIST (https://webbook.nist.gov/, accessed on 20 April 2025)). All 12 flavonoid standards, namely, apigenin (ST00410120), eriodictyol (ST16250120), kaempferol (ST00450120), chrysoeriol (STC5610120), isorhamnetin (ST03200120), sinensetin (ST16210120), gardenin B (ST81990120), quercitrin (ST01810120), diosmin (ST05830120), naringin (ST00210120), hesperetin (ST06790120), and hesperidin (ST01300120), were obtained from Shanghai Standard Technology Co., Ltd. (Shanghai, China). Raw LC-MS data were processed using MS-DIAL (version 4.24) for normalization and the m/z values, RT, and corresponding intensities were subsequently exported for multivariate analysis. For each feature, the representative ion ([M+H]^+^) was selected as the one with the highest intensity and most consistent chromatographic shape. Features were only retained if they exhibited a clear chromatographic peak (peak width at half height > 5 s) and a signal-to-noise ratio greater than 10. Furthermore, potential isobaric overlaps were minimized by setting a high mass accuracy threshold of ±5 ppm for peak extraction.

### 2.4. Statistical Analysis

Raw multiple reaction monitoring data were processed using MassHunter Workstation v10.0 for metabolite identification based on RT and m/z, excluding isotopic peaks, and compound identities were subsequently confirmed using authentic standards. For data visualization, LC-MS data were converted to Abf format and normalized in MS-DIAL 4.24, generating a three-dimensional matrix of m/z, RT, and signal intensity. The relative standard deviation (RSD) values ranging from 0.71% to 4.82% confirmed the robustness, precision, and reliability of this analytical method.

Cluster heatmaps and PCA were performed using MetaboAnalyst 6.0 (https://www.metaboanalyst.ca/, accessed on 5 May 2026). Heatmaps were generated based on metabolite abundance values from five biological replicates per cultivar at each developmental stage, with hierarchical clustering performed using Euclidean distance and Ward’s linkage method. The S-plot of OPLS-DA models and permutation tests of PLS-DA models were generated using SIMCA software (version 13.0; Umetrics, Umea, Sweden). Prior to parametric testing, the normality of data distribution was assessed using the Shapiro–Wilk test. For comparisons between two groups, Student’s *t*-test was employed to calculate *p-*values. Metabolites with FDR-adjusted *p* < 0.05 were considered statistically significant. Furthermore, metabolites were defined as significantly regulated between groups when the variable importance in projection (VIP) score was >1, the absolute log_2_ fold change (|log_2_FC|) was >1, and *p* < 0.05.

## 3. Results

### 3.1. Characterization of Flavonoids in STJ and DLJ

To reveal the dynamic changes in flavonoids within STJ and DLJ during fruit development, LC-MS/MS-based metabolomics was applied to profile flavonoid compositions in both peel and flesh across six maturation phases. The total ion chromatogram, relative contents, integral values, and putative identifications are summarized in Supplementary Figure S1A and Table S1. A total of 205 presumably annotated flavonoids were systematically classified into seven structural subclasses: chalcone (1), dihydroflavonols (3), flavonoid C-glycosides (11), flavanones (25), flavones (144), flavonol glycosides (20), and isoflavanones (1) (Figure S1B). Among these, 12 flavonoids were definitively identified.

### 3.2. Dynamic Changes in Flavonoids in Peel and Flesh at Different Development Stages

PCA revealed clear compositional divergence in flavonoid metabolites among the four sample groups—SP (STJ peel), DP (DLJ peel), SF (STJ flesh), and DF (DLJ flesh)—across successive developmental stages (Figure 1B–E). Specifically, SP samples from stages 1 to 4 (SP-1 in July, SP-2 in August, SP-3 in September, and SP-4 in October) exhibited distinct separation, indicating substantial metabolic differences during these early developmental periods. In contrast, samples from stages 5 and 6 (SP-5 in November and SP-6 in December) formed tight clusters, reflecting relatively minor metabolic changes compared to the preceding phases. A similar trend was observed in DP samples: stage 1 was significantly separated from the rest, whereas stages 2 through 6 showed minimal separation, suggesting a marked shift in the flavonoid profile of DLJ peel between stage 1 and the subsequent stages. The early developmental stages of the flesh tissues (SF-1 to SF-3 and DF-1 to DF-3) exhibited substantial variation compared to the later stages. Moreover, the temporal variations in four tissues of the two citrus species were compared during the same period (Figure S2A–F). The results revealed that SP and DP exhibited distinct separation across almost all stages, with the exception of stage 5, whereas SF and DF displayed minimal differentiation, rendering them largely indistinguishable throughout the maturation process.

**Figure 2:**
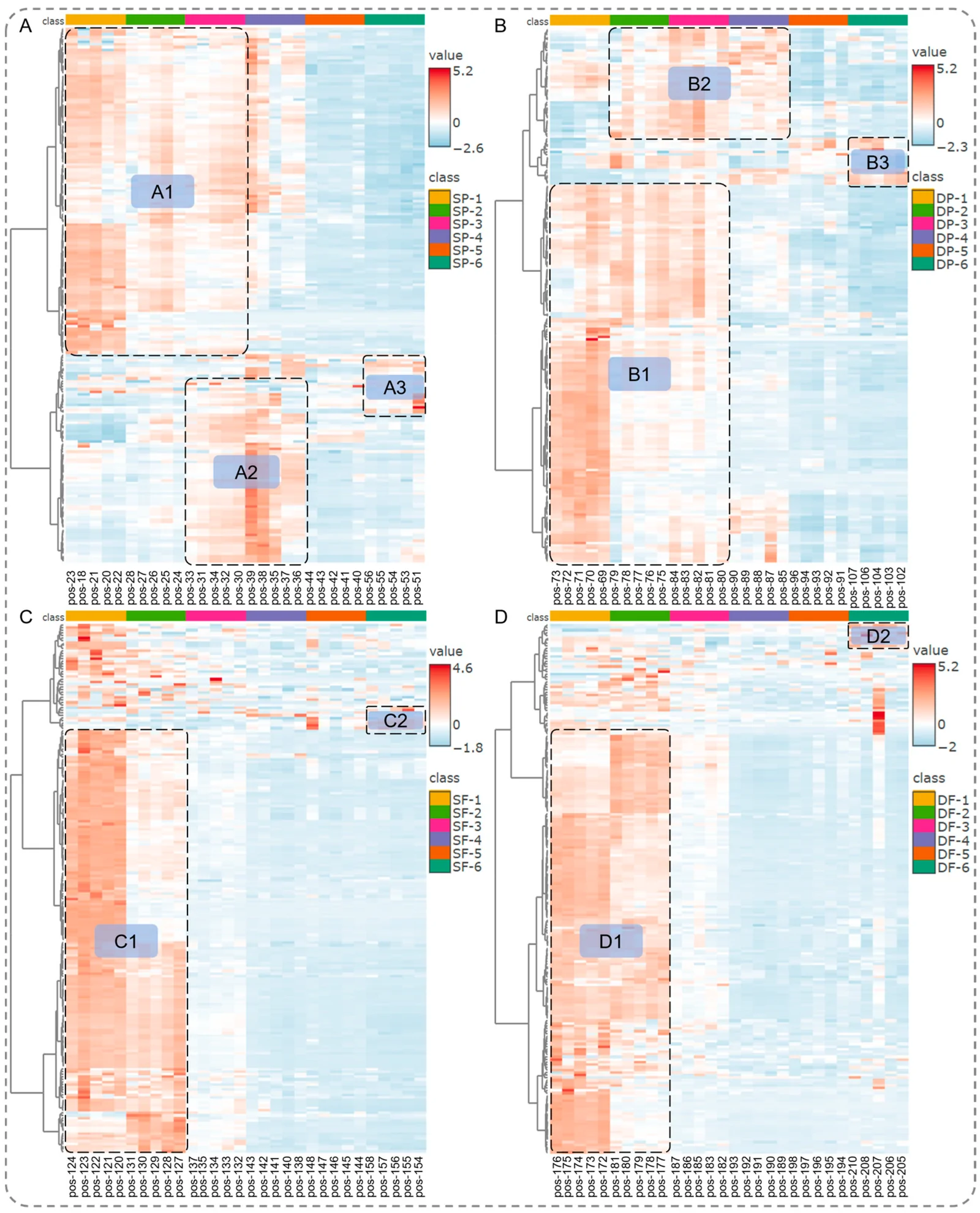
Heatmap showing the clustering of identified metabolites in SP (**A**), DP (**B**), SF (**C**), and DF (**D**) at different development stages. Dashed boxes labeled **A**, **B**, **C**, and **D** delineate distinct clusters of structurally related flavonoids.

Furthermore, the heatmap revealed dynamic accumulation patterns of flavonoid metabolites across the different developmental stages. In SP (Figure 2A and Figure S3A), flavonoid compounds in group A1, such as salvigenin, isorhamnetin, naringenin, naringin, diosmetin, luteolin, eriodictyol, cirsimaritin, and quercitrin, exhibited the highest concentrations during the initial stage (SP-1) and then subsequently decreased during SP-2 and SP-3. A distinct subgroup, A2, including rutin, tangeretin, sinensetin, hesperidin, demethylnobiletin, gardenin B, nobiletin, and orientin, accumulated significantly during SP-3 and SP-4, and subsequently underwent marked depletion during the later maturation phases (SP-5 and SP-6). Compounds in subgroup A3, namely, narirutin, kaempferitrin, and pinobanksin, were significantly enriched during SP-6. Additionally, the temporal accumulation patterns of flavonoids in subgroups B1, B2, and B3 in DP closely mirrored those of subgroups A1, A2, and A3 in SP, respectively (Figure 2B and Figure S3B).

Notably, the accumulation of specific flavonoids, including 5,6,3′-trihydroxy-3,7,4′-trimethoxyflavone, demethylnobiletin, kaempferide, gardenin B, chrysoeriol, and 5-hydroxy-6,7,3′,4′,5′-pentamethoxyflavone, peaked exclusively at the SP-4 in STJ, whereas no comparable temporal accumulation pattern was observed in DLJ. Such spatiotemporal accumulation highlights a cultivar-specific metabolic divergence, warranting further investigation to elucidate the underlying transcriptional regulatory networks driving this disparity. Additionally, SF and DF exhibited congruent temporal patterns in flavonoid metabolism (Figure 2C,D and Figure S3C,D). Both tissues maintained relatively high abundances of flavonoid metabolites during the first two periods, followed by progressive diminution of compound concentrations from the third period onward.

### 3.3. Differential Analysis of Flavonoids in Peel and Flesh at Different Development Stages

OPLS-DA was employed to identify characteristic metabolites during maturation in STJ and DLJ. The robustness and predictive accuracy of the OPLS-DA models were rigorously evaluated through 7-fold cross-validation, yielding the goodness-of-fit (R^2^Y) and predictive ability (Q^2^) values. As detailed in Figures S4 and S5 and Table S2, the R^2^Y and Q^2^ values across the four tissues at various developmental stages ranged from 0.985 to 0.999 and 0.964 to 0.996, respectively, demonstrating high model reliability and predictive performance.

**Figure 3:**
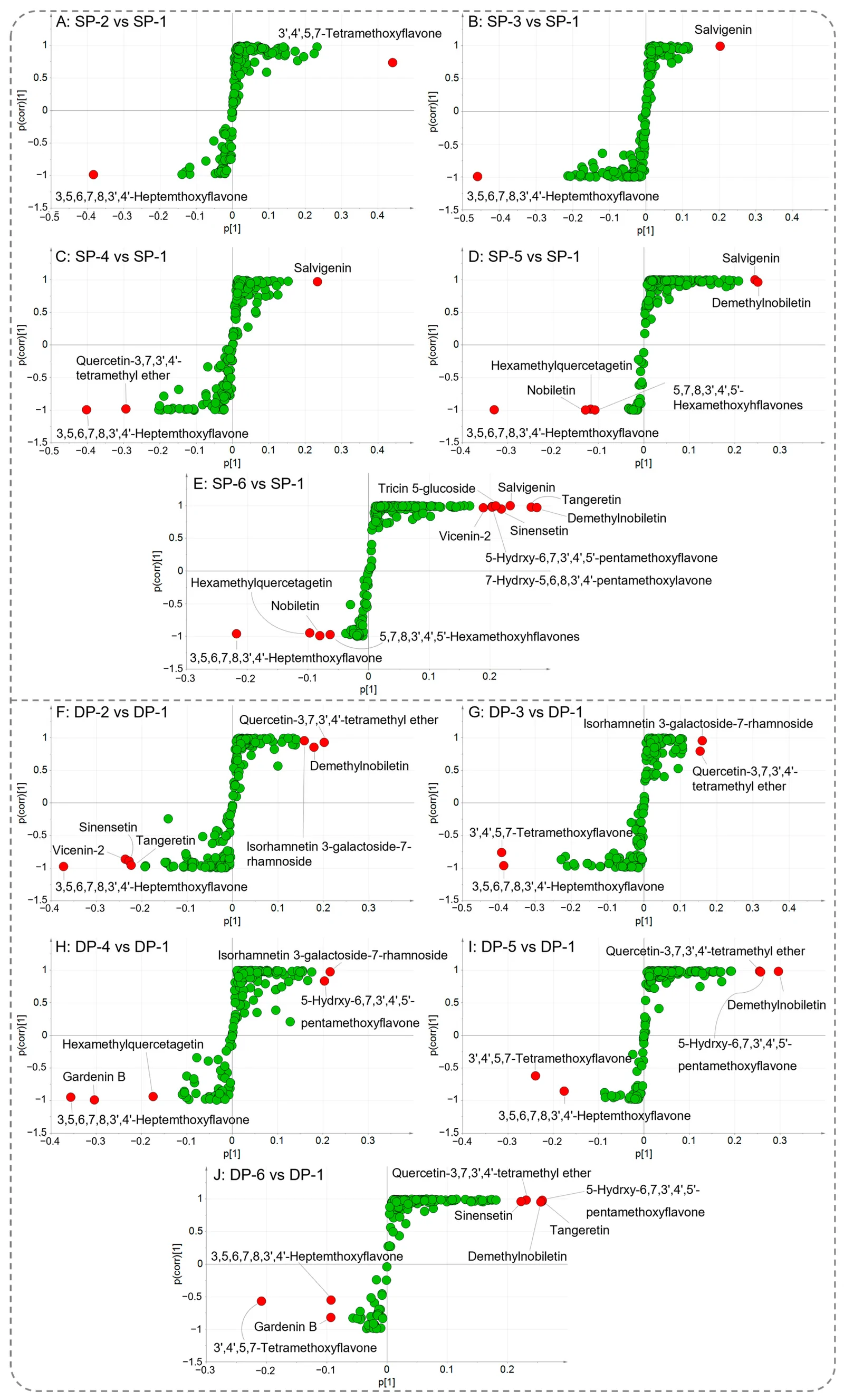
OPLS-DA analysis of the flavonoids in all comparison groups of SP and DP. (**A**) SP-2 vs. SP-1; (**B**) SP-3 vs. SP-1; (**C**) SP-4 vs. SP-1; (**D**) SP-5 vs. SP-1; (**E**) SP-6 vs. SP-1; (**F**) DP-2 vs. DP-1; (**G**) DP-3 vs. DP-1; (**H**) DP-4 vs. DP-1;(**I**); DP-5 vs. DP-1; (**J**) DP-6 vs. DP-1.

S-plot analysis revealed that the flavonoids located in the extremities of the S-curve distribution could be regarded as the differential metabolites [14]. As illustrated in Figure 3, the key discriminating metabolites in SP included salvigenin, demethylnobiletin, and nobiletin, whereas sinensetin, tangeretin, and 3′,4′,5,7-tetramethoxyflavone were identified as the predominant flavonoids in DP. The prominence of these polymethoxylated flavonoids (PMFs) highlights their potential role as biochemical markers for varietal differentiation. Interestingly, 3,5,6,7,8,3′,4′-heptamthoxyflavone (HPMF) was identified as a differential metabolite across all comparison groups, revealing its significant role in citrus peel maturation. Additionally, a higher number of PMFs were detected in the comparison groups of SP-1 vs. SP-6 and DP-1 vs. DP-6 (Figure 3E,J), compared with other comparative groups. This suggests that PMFs might be the key metabolites of the peel maturation process of both STJ and DLJ.

In addition, significant variations in flavonoid composition were observed in the flesh tissues (Figures S5 and S6). With the exception of SF-1 vs. SF-2 and DF-1 vs. DF-2, the differential metabolites identified in the remaining pairwise comparisons for both SF and DF predominantly consisted of sinensetin, 3′,4′,5,7-tetramethoxyflavone, vicenin-2, tangeretin, naringin, naringenin, and demethylnobiletin. Notably, there was no significant change in the content and composition of flavonoids in flesh from the SF-3 stage to maturity, which is consistent with the result of the heatmap analysis in Figure 2.

To reconcile the discrepancies between the OPLS-DA and PLS-DA models, a rigorous multi-criteria filtering strategy was implemented to ensure model robustness. Specifically, the most robust discriminant features were selected by intersecting three validation parameters: (1) a VIP score > 1 shared by both the OPLS-DA and PLS-DA models, (2) a FDR-adjusted *p*-value < 0.05 from univariate analysis, and (3) an extreme value in the S-plot (|p(corr)| > 0.5). The finalized list of robust discriminant metabolites is presented in Supplementary Table S3. To mitigate the risk of overfitting potentially introduced by small sample sizes in multivariate models, 200 response permutation tests were performed for each pairwise comparison via PLS-DA. PLS-DA model was considered statistically valid when the original Q^2^ value exceeded 0.9 and the Q^2^ intercept from permutation tests was negative, demonstrating that the original model exhibited significantly better predictive performance than permuted models. All the comprehensive validation metrics—including R^2^Y, Q^2^, permutation test intercepts, and their corresponding *p*-values—are detailed in Supplementary Table S2 and Figures S7 and S8.

### 3.4. Differential Analysis of Flavonoids in Two Tissues Between STJ and DLJ

**Figure 4:**
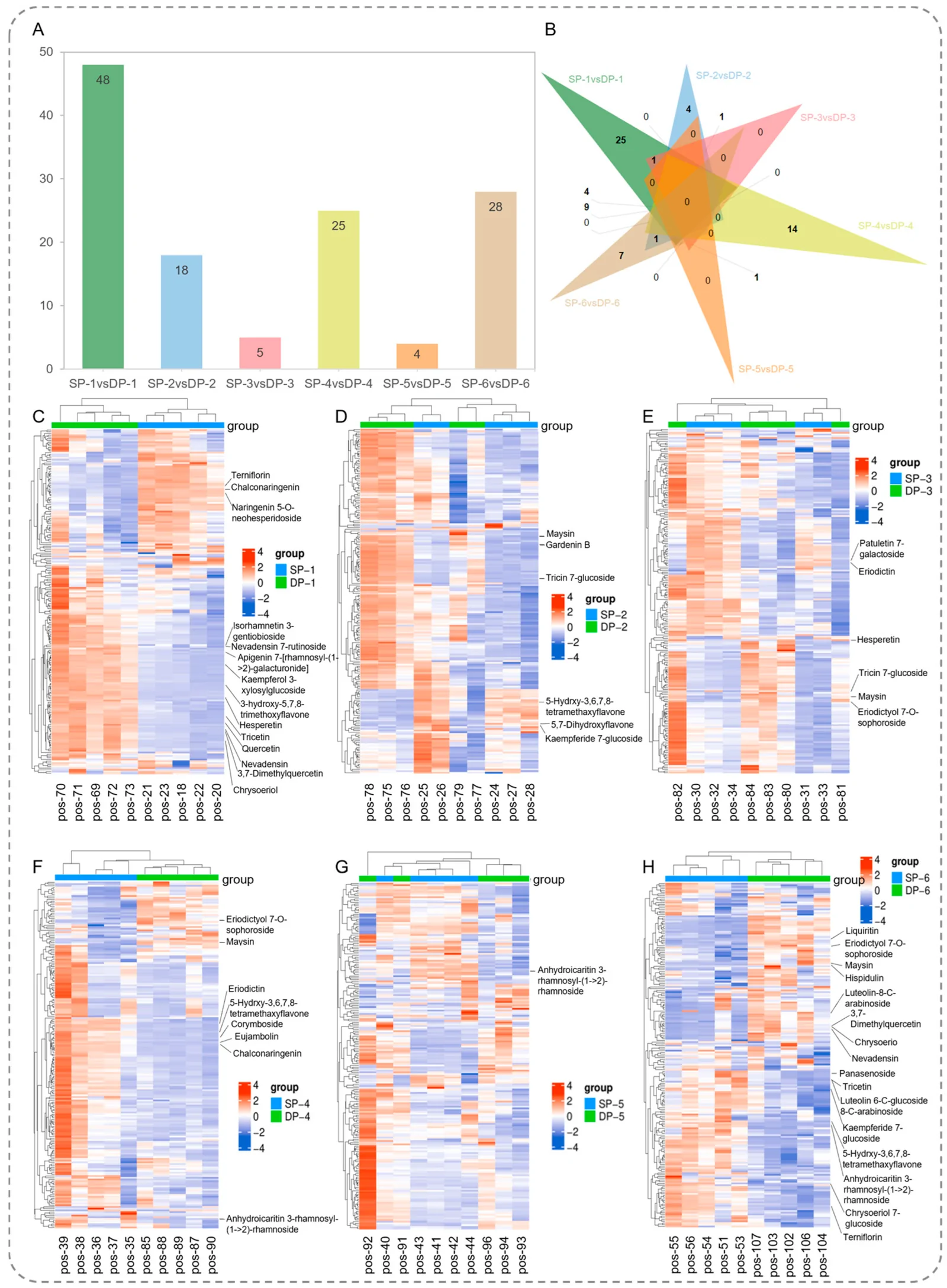
Differential metabolic analysis of SP and DP across six developmental stages. (**A**) Numbers of differential metabolites in each comparison. (**B**) Venn of the number of differential metabolites in the six period comparisons. (**C**–**H**) Metabolite clustering analysis of samples from each period of SP vs. DP: (**C**) SP-1 vs. DP-1; (**D**) SP-2 vs. DP-2; (**E**) SP-3 vs. DP-3; (**F**) SP-4 vs. DP-4; (**G**) SP-5 vs. DP-5; (**H**) SP-6 vs. DP-6.

Heatmap analysis of flavonoids was performed for the comparisons of SP vs. DP and SF vs. DF across six developmental stages (Figure 4C–H). Cluster analysis revealed distinct separation patterns among the 12 comparison groups, with the heatmap visualization further exposing a substantial number of differentially accumulated flavonoids. These discriminant metabolites were identified as critical biomarkers contributing to the chemotaxonomic distinction between SP vs. DP and SF vs. DF, respectively. Furthermore, comparative metabolomic profiling across four tissues was performed using stringent statistical criteria: *p* < 0.05, VIP scores > 1.0, and |log2FC| ≥ 1.0. The results showed that 81 common differential metabolites in SP vs. DP and 66 consensus differential metabolites in SF vs. DF were identified (Table S4). Specifically, 48 metabolites were identified in SP-1 vs. DP-1, followed by 18 in SP-2 vs. DP-2, 5 in SP-3 vs. DP-3, 25 in SP-4 vs. DP-4, 4 in SP-5 vs. DP-5, and 28 in SP-6 vs. DP-6 (Figure 4A). In addition, 27 metabolites in SF-1 vs. DF-1, 12 in SF-2 vs. DF-2, 5 in SF-3 vs. DF-3, 25 in SF-4 vs. DF-4, 5 in SF-5 vs. DF-5, and 13 in SF-6 vs. DF-6 (Figure S9A).

Venn diagram analysis of the differential metabolites revealed an absence of shared compounds across all comparison groups (Figure 4B), indicating profound compositional differences between STJ and DLJ, especially in the comparisons of SP-1 vs. DP-1, SP-4 vs. DP-4, SP-6 vs. DP-6 and SF-1 vs. DF-1, SF-4 vs. DF-4, SF-6 vs. DF-6. Furthermore, several metabolites were uniquely identified in specific comparison groups. For instance, 25 unique differential metabolites were identified in SP-1 vs. DP-1, such as quercetin, and hesperetin. In the comparison of SP-2 vs. DP-2, four unique differential metabolites were found: clematin, luteolin 3-O-(6′′-malonylglucoside), sakuranetin, and gardenin B. Additionally, 14 unique differential metabolites were identified in SP-4 vs. DP-4, such as narirutin, naringin, and naringenin. In SP-6 vs. DP-6, seven unique differential metabolites were identified: chrysoeriol 7-glucoside, 6-hydroxymyricetin 3,6,3′,5′-tetramethyl ether 7-glucoside, luteolin-8-C-arabinoside, 6′′-O-(3-hydroxy-3-methylglutaroyl) astragalin, 5,6-dihydroxy-7,8,3′,4′-tetramethoxyflavone, liquiritin, and hispidulin. Similarly, about 19, 10, 2, 16, 1, and 9 differential metabolites in SF and DF specific to certain comparison groups were also identified, respectively (Figure S9). These results provide valuable insights for future studies aiming to elucidate the dynamic variations in flavonoids in STJ and DLJ during fruit maturation.

### 3.5. Dynamic Changes in Metabolites in Flavonoid Biosynthesis Pathway

**Figure 5:**
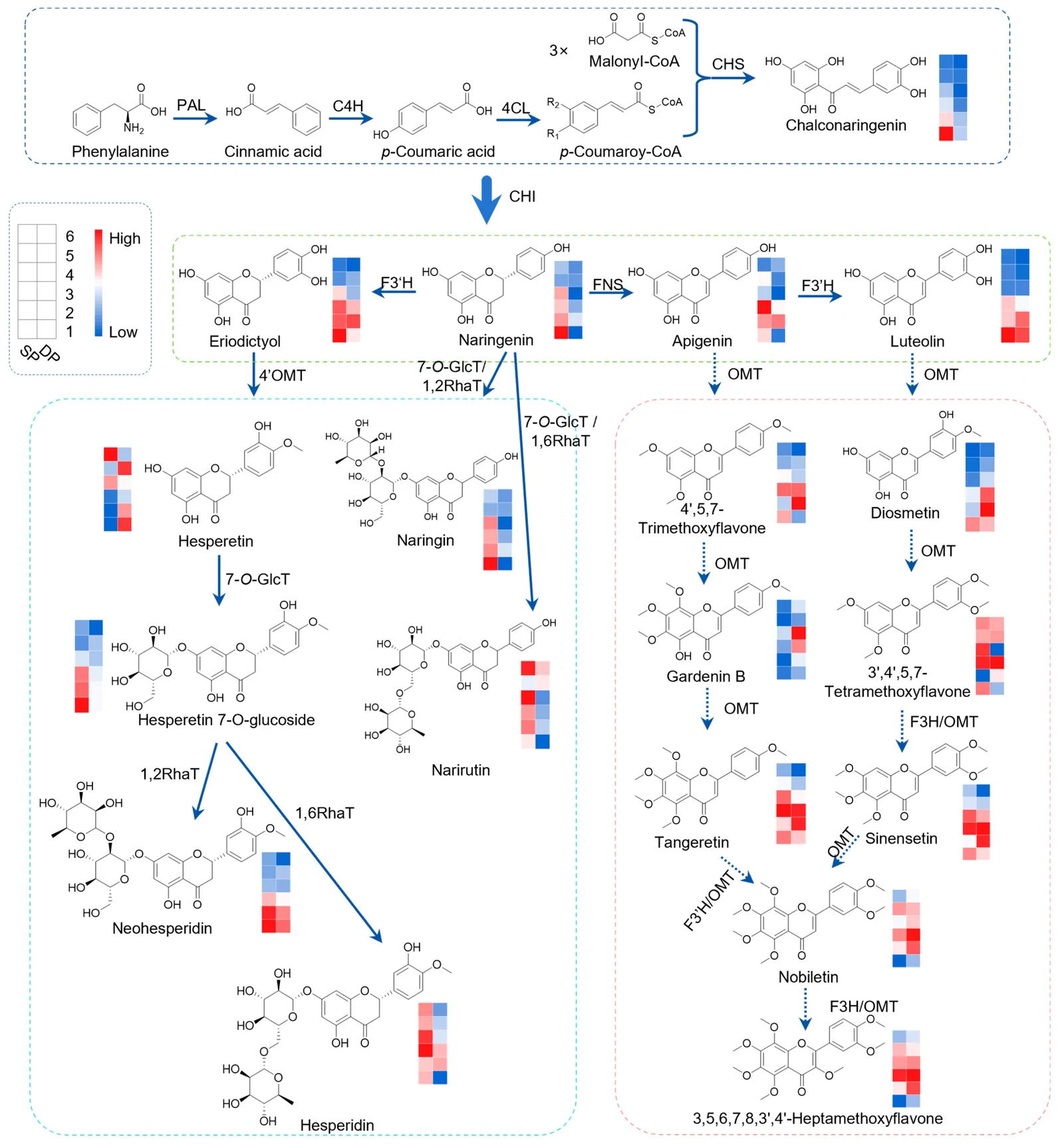
Dynamic changes in metabolites in flavonoid biosynthesis pathway. *PAL*: phenylalanine ammonialyase; *C4H*: cinnamate 4-hydroxylase; *4CL*: 4-coumarate:CoA ligase; *CHS*: chalcone synthesis; *F3′H*: flavonoid 3′-hydroxylase; *FNS*: flavone synthase; *F3H*: flavanone 3-hydroxylase; *OMT*: O-methyltransferase; *7-O-GlcT*: 7-O-glucosyltransferase; *1,2RhaT*: 1,2 rhamnosyltransferase; *1,6RhaT*: 1,6 rhamnosyltransferase.

Over 80 flavonoids, categorized into six major subclasses such as flavones and flavonols, have been identified in *Citrus*. The structural diversification of flavonoid skeletons—through processes including hydroxylation, methylation, and glycosylation—plays a critical role in determining their bioactivity, metabolic stability, and ecological fitness [19,20]. In the present study, comparative metabolomics revealed significant differences in both the content and composition of flavonoids between STJ and DLJ. To further elucidate these differences, the identified crucial flavonoids were integrated into simplified metabolic pathway maps, as illustrated in Figure 5.

Hydroxylation is pivotal for the structural diversification of flavonoids, as the site-specific introduction of hydroxyl groups enables subsequent glycosylation and methylation modifications [21]. Glycosylation is a crucial strategy for enhancing the stability of flavonoids [22]. The most representative processes involve the glycosylation of hesperetin and naringenin, which is presumably driven by the differential expression of *1,6RhaT* and *1,2RhaT*, generating glycoside derivatives with potentially opposing biochemical properties [23,24]. In this study, the contents of neohesperidin, hesperidin, naringin and narirutin in SP were significantly higher than those in DP, indicating that a divergent regulatory mechanism of flavonoid glycosylation between the two cultivars.

Methylation is a critical modification of flavonoids in citrus [25], especially for PMFs. PMFs exhibit tissue-specific biosynthesis characteristics in citrus plants, accumulating exclusively in the peel where they play multifunctional roles in both plant defense and human health [26]. In this study, the differential accumulated PMFs identified via S-plot analysis serve as discriminatory biomarkers for distinguishing SP and DP. This suggests that the biosynthetic pathways of flavonoids in SP and DP are governed by divergent regulatory mechanisms, and the dynamic accumulation of flavonoids is closely associated with tissue development and environmental adaptation [27,28]. Therefore, further research to elucidate the genes and their expression profiles regulating these biosynthesis pathways is essential.

## 4. Discussion

STJ and DLJ are economically important crops cultivated in southern China [9,10]. Flavonoids, as crucial bioactive components of these citrus fruits, exhibit notable antiviral and anticancer properties [29,30]. However, our understanding of the dynamic changes in flavonoid profiles across various developmental stages in STJ and DLJ remains limited. Metabolomics serves as a fundamental and widely employed method for elucidating differences in secondary metabolites in plants [31]. Therefore, in the present study, we utilized a metabolomic approach based on LC-MS/MS to comprehensively map dynamic changes in flavonoids in the peel and flesh of STJ and DLJ across six developmental stages.

### 4.1. Developmental Dynamics and Biological Significance of Flavonoid Components

The PCA score plots (Figure 1B–E) and the heatmap of peel flavonoid profiles (Figure 2A,B) revealed a clear temporal trajectory, wherein flavonoids from the early developmental stages (stages 1–4) remained distinctly separated, whereas those from the late maturation stages (stages 5–6) converged into a tight cluster. This pattern indicates that the flavonoids in the peel undergo a fundamental metabolic transition from a highly dynamic state to a more homeostatic condition at ripening completion [32]. The results reveal a pronounced tissue-specific and developmental-dependent flavonoid accumulation pattern in the peels, compared to the relatively stable profile observed in the flesh. The temporal shifts in flavonoids in the peel—from early-stage flavanones (e.g., naringenin and naringin) to mid-stage PMFs and flavonoid glycoside (e.g., tangeretin, sinensetin, nobiletin, demethylnobiletin and hesperidin)—likely reflect a dynamic remodeling of defense mechanisms adapted to fruit maturation [33]. The accumulation of PMFs in the peel reinforces its role as a physiological barrier, with tangeretin and nobiletin contributing direct antimicrobial defense [34,35,36]. Concurrently, the high abundance of hesperidin synergistically enhances antioxidant and anti-inflammatory capacities, establishing a multifaceted chemical defense system against biotic and abiotic stresses [37]. As the fruit proceeds to late maturation stage (stages 5–6), the metabolic demand for these defensive metabolites diminishes, leading to their catabolism or conversion, which aligns with the observed decline and the achievement of metabolic homeostasis characteristic of ripened peel. Notably, the specific enrichment of PMFs at the SP-4 stage in STJ implied a cultivar-specific transcriptional activation, potentially driven by the upregulation of OMT and flavonoid hydroxylases [38], which requires further experimental validation.

A particularly striking finding of the S-plot analysis was that HPMF was consistently identified as a differential metabolite across all peel comparison groups, irrespective of cultivar or developmental stage, suggesting that it acts as a universal molecular indicator of overall peel maturation. However, despite this ubiquitous differential abundance, HPMF likely exhibits a unidirectional, monotonic accumulation trajectory rather than stage-specific peaks, thereby diminishing its discriminatory capacity for demarcating specific developmental stage. Furthermore, the most pronounced PMF differentials were observed in the comparisons of SP-1 vs. SP-6 and DP-1 vs. DP-6, underscoring the centrality of PMF biosynthesis throughout the entire maturation process.

### 4.2. Specific Metabolic Characteristics Between STJ and DLJ

The comprehensive comparison of SP vs. DP and SF vs. DF (Figure 4 and Figure S9) identified 81 and 66 common differential metabolites, respectively, yet the Venn diagram analysis revealed no shared differential metabolites across all stages. This result implied that metabolic discrimination between these tissues is highly stage-dependent and the domestication has differentially shaped the flavonoid biosynthetic machinery in STJ and DLJ. Most notably, the mature stage (SP-6 vs. DP-6) exhibited a specific enrichment of unique differential metabolites (Table S4), including liquiritin, hispidulin, chrysoeriol 7-glucoside, 6-hydroxymyricetin 3,6,3′,5′-tetramethyl ether 7-glucoside, luteolin-8-C-arabinoside, 6′′-O-(3-hydroxy-3-methylglutaroyl) astragalin, and 5,6-dihydroxy-7,8,3′,4′-tetramethoxyflavone. Several of these compounds have been previously reported to possess anticancer [39], antitumor, and anti-inflammatory capacity [40]. Although our current study is limited to their metabolic profiling, this stage-specific accumulation provides a metabolomic foundation for the potential health-promoting properties of these cultivars and points to the need for future functional assays.

Furthermore, higher levels of naringin and its aglycone naringenin were observed in SP-4 compared to DP-4. Previous studies have indicated that these compounds are potentially regulated by *1,2RhaT* [23,41] and play critical roles in plant chemical defense mechanisms due to their inherent bitterness [42]. Although the present study is primarily confined to the accumulation of these metabolites, future research should focus on elucidating the functional applications in the context of fruit quality regulation.

## 5. Conclusions

The present study compared the dynamic accumulation patterns of flavonoids in the peel and flesh of STJ and DLJ during the developmental stages. Multivariate statistical analysis indicated significant divergence in flavonoid content between SP and DP. Notably, compared to DP, SP exhibited elevated levels of neohesperidin, hesperidin, naringin, and narirutin. These findings provide a robust theoretical foundation for understanding the dynamic changes in flavonoids during the developmental stages of STJ and DLJ, and contribute to the elucidation of the metabolic differences in flavonoids between STJ and DLJ.

## Data Availability

The original contributions presented in this study are included in the article/Supplementary Material. Further inquiries can be directed to the corresponding authors.

## Supplementary Materials

The following supporting information can be downloaded at: https://www.mdpi.com/article/10.3390/cimb48080774/s1.

## Abbreviations

STJ	*Citrus reticulata* ‘Shiyue Ju’	*1,2RhaT*	1,2 Rhamnosyltransferase
DLJ	*C. reticulata* ‘Denglong Ju’	*FNS*	Flavone synthase
SP	The peel of STJ	*1,6RhaT*	1,6 Rhamnosyltransferase
SF	The flesh of STJ	*F3′H*	Flavanone-3′-hydroxylase
DP	The peel of DLJ	PCA	Principal component analysis
DF	The flesh of DLJ	OPLS-DA	Orthogonal partial least squares-discriminant analysis
*PAL*	Phenylalanine ammonialyase	RT	Retention time
*C4H*	Cinnamate 4-hydroxylase	VIP	Variable importance in project
*4CL*	4-coumarate:CoA ligase	PMFs	Polymethoxylated flavonoids
*CHS*	Chalcone synthase	HPMF	3,5,6,7,8,3′,4′-Heptemthoxyflavone
*7-O-GlcT*	7-O-Glucosyltransferase		

## References

[1] Ferreira S.S., Silva A.M., Nunes F.M. (2018). Citrus reticulata Blanco peels as a source of antioxidant and anti-proliferative phenolic compounds. Ind. Crop Prod..

[2] Liu N., Li X., Zhao P., Zhang X., Qiao O., Huang L., Guo L., Gao W. (2021). A review of chemical constituents and health-promoting effects of citrus peels. Food Chem..

[3] Saini R.K., Ranjit A., Sharma K., Prasad P., Shang X., Gowda K.G.M., Keum Y. (2022). Bioactive compounds of Citrus fruits: A review of composition and health benefits of carotenoids, flavonoids, limonoids, and terpenes. Antioxidants.

[4] Shamsudin N.F., Ahmed Q.U., Mahmood S., Ali S.S., Khatib A., Mukhtar S., Alsharif M.A., Parveen H., Zakaria Z.A. (2022). Antibacterial Effects of Flavonoids and Their Structure-Activity Relationship Study: A Comparative Interpretation. Molecules.

[5] Zhao C., Wang F., Lian Y., Xiao H., Zheng J. (2020). Biosynthesis of citrus flavonoids and their health effects. Crit. Rev. Food Sci..

[6] Ben Hsouna A., Sadaka C., Generalić Mekinić I., Garzoli S., Švarc-Gajić J., Rodrigues F., Morais S., Moreira M.M., Ferreira E., Spigno G. (2023). The Chemical Variability, Nutraceutical Value, and Food-Industry and Cosmetic Applications of Citrus Plants: A Critical Review. Antioxidants.

[7] Wu G.A., Terol J., Ibanez V., López-García A., Pérez-Román E., Borredá C., Domingo C., Tadeo F.R., Carbonell-Caballero J., Alonso R. (2018). Genomics of the origin and evolution of Citrus. Nature.

[8] Zhang L., Chen D., Lü G. (2023). Breeding report of a new Citrus reticulata Blanco cultivar Shatang Lantern tangerine. J. Fruit Sci..

[9] Liu G., Ping W., Ling D., Li J., Zhang L., Li L., Zhang Z., Zhang L. (2023). Composition and activity of peel polysaccharides from three citrus fruits. Food Sci. Technol..

[10] Cao J., Kang C., Chen Y., Karim N., Wang Y., Sun C. (2020). Physiochemical changes in *Citrus reticulata* cv. Shatangju fruit during vesicle collapse. Postharvest Biol. Technol..

[11] Juan T., Feiyong W., Lan Z. (2023). Transcriptome analysis based on character difference between Shatang tangerine and Lantern Shatang tangerine. J. Fruit Sci..

[12] Shen S., Zhan C., Yang C., Fernie A.R., Luo J. (2023). Metabolomics-centered mining of plant metabolic diversity and function: Past decade and future perspectives. Mol. Plant.

[13] Fayek N.M., Farag M.A., Saber F.R. (2021). Metabolome classification via GC/MS and UHPLC/MS of olive fruit varieties grown in Egypt reveal pickling process impact on their composition. Food Chem..

[14] Wang C., Han F., Chen X., Zhao A., Wang D. (2022). Time-series based metabolomics reveals the characteristics of the color-related metabolites during the different coloration stages of *Zanthoxylum bungeanum* peel. Food Res. Int..

[15] Shen F., Wang T., Zhang R., Zhong B., Wu Z. (2024). Metabolism and release of characteristic components and their enzymatic mechanisms in *Pericarpium Citri Reticulatae* co-fermentation. Food Chem..

[16] Wang A., Li R., Ren L., Gao X., Zhang Y., Ma Z., Ma D., Luo Y. (2018). A comparative metabolomics study of flavonoids in sweet potato with different flesh colors (*Ipomoea batatas* (L.) Lam). Food Chem..

[17] Shi J., Simal-Gandara J., Mei J., Ma W., Peng Q., Shi Y., Xu Q., Lin Z., Lv H. (2021). Insight into the pigmented anthocyanins and the major potential co-pigmented flavonoids in purple-coloured leaf teas. Food Chem..

[18] Guan Q., Huang D., Zhang L., Lv Z., Chen W. (2025). Integrated metabolomics and transcriptomics reveal the tissue-specific distribution patterns and potential molecular mechanisms in flavonoids between *Citrus reticulata* ‘Shiyue Ju’ and its mutant *C. reticulata* ‘Denglong Ju’. Sci. Hortic..

[19] Tang S., Wang B., Liu X., Xi W., Yue Y., Tan X., Bai J., Huang L. (2025). Structural insights and biological activities of flavonoids: Implications for novel applications. Food Front..

[20] Shen N., Wang T., Gan Q., Liu S., Wang L., Jin B. (2022). Plant flavonoids: Classification, distribution, biosynthesis, and antioxidant activity. Food Chem..

[21] Halbwirth H. (2010). The Creation and Physiological Relevance of Divergent Hydroxylation Patterns in the Flavonoid Pathway. Int. J. Mol. Sci..

[22] Xiao J., Muzashvili T.S., Georgiev M.I. (2014). Advances in the biotechnological glycosylation of valuable flavonoids. Biotechnol. Adv..

[23] Chen J., Yuan Z., Zhang H., Li W., Shi M., Peng Z., Li M., Tian J., Deng X., Cheng Y. (2019). *Cit1,2RhaT* and two novel *CitdGlcTs* participate in flavor-related flavonoid metabolism during citrus fruit development. J. Exp. Bot..

[24] Chen J., Qiu X., Sun Z., Luan M., Chen J. (2024). Genome-wide analysis of UDP-glycosyltransferase family in Citrus sinensis and characterization of a UGT gene encoding flavonoid 1–2 rhamnosyltransferase. Int. J. Biol. Macromol..

[25] Koirala N., Thuan N.H., Ghimire G.P., Thang D.V., Sohng J.K. (2016). Methylation of flavonoids: Chemical structures, bioactivities, progress and perspectives for biotechnological production. Enzym. Microb. Technol..

[26] Peng Q., Zhang Y., Zhu M., Bao F., Deng J., Li W. (2024). Polymethoxyflavones from citrus peel: Advances in extraction methods, biological properties, and potential applications. Crit. Rev. Food Sci..

[27] Singh A.K., Dhanapal S., Yadav B.S. (2020). The dynamic responses of plant physiology and metabolism during environmental stress progression. Mol. Biol. Rep..

[28] Treutter D. (2006). Significance of flavonoids in plant resistance: A review. Environ. Chem. Lett..

[29] Alam F., Mohammadin K., Shafique Z., Amjad S.T., Asad M.H.H.B. (2022). Citrus flavonoids as potential therapeutic agents: A review. Phytother. Res..

[30] Wang Y., Qian J., Cao J., Wang D., Liu C., Yang R., Li X., Sun C. (2017). Antioxidant Capacity, Anticancer Ability and Flavonoids Composition of 35 Citrus (*Citrus reticulata* Blanco) Varieties. Molecules.

[31] Salam U., Ullah S., Tang Z., Elateeq A.A., Khan Y., Khan J., Khan A., Ali S. (2023). Plant Metabolomics: An Overview of the Role of Primary and Secondary Metabolites against Different Environmental Stress Factors. Life.

[32] Liu H., Long C., Wang S., Fu X., Dong J., Yan W., Dong M., Duan M., Zhang B., Yang H. (2025). Combined metabolome and transcriptome profiling provides insights into dynamic molecular control of lemon (*Citrus Limon* L.) peel development. BMC Plant Biol..

[33] Gaikwad P.N., Sidhu G.S., Brar N.S., Singh J., Tokala V.Y., Sharma A., Manchanda P. (2025). Roles of metabolites in fruit maturation, HLB-defense regulation and crosstalk between phytohormone signalling pathways in citrus. Plant Growth Regul..

[34] Peng Z., Zhang H., Li W., Yuan Z., Xie Z., Zhang H., Cheng Y., Chen J., Xu J. (2021). Comparative profiling and natural variation of polymethoxylated flavones in various citrus germplasms. Food Chem..

[35] Zhuang W., Li Y., Shu X., Pu Y., Wang X., Wang T., Wang Z. (2023). The Classification, Molecular Structure and Biological Biosynthesis of Flavonoids, and Their Roles in Biotic and Abiotic Stresses. Molecules.

[36] Yao X., Zhu X., Pan S., Fang Y., Jiang F., Phillips G.O., Xu X. (2012). Antimicrobial activity of nobiletin and tangeretin against Pseudomonas. Food Chem..

[37] Parhiz H., Roohbakhsh A., Soltani F., Rezaee R., Iranshahi M. (2015). Antioxidant anti-inflammatory properties of the citrus flavonoids hesperidin hesperetin: An updated review of their molecular mechanisms experimental models. Phytother. Res..

[38] Wen J., Wang Y., Lu X., Pan H., Jin D., Wen J., Jin C., Sahu S.K., Su J., Luo X. (2024). An integrated multi-omics approach reveals polymethoxylated flavonoid biosynthesis in *Citrus reticulata* cv. Chachiensis. Nat. Commun..

[39] Liu K., Zhao F., Yan J., Xia Z., Jiang D., Ma P. (2020). Hispidulin: A promising flavonoid with diverse anti-cancer properties. Life Sci..

[40] Qin J., Chen J., Peng F., Sun C., Lei Y., Chen G., Li G., Yin Y., Lin Z., Wu L. (2022). Pharmacological activities and pharmacokinetics of liquiritin: A review. J. Ethnopharmacol..

[41] Frydman A., Weisshaus O., Bar-Peled M., Huhman D.V., Sumner L.W., Marin F.R., Lewinsohn E., Fluhr R., Gressel J., Eyal Y. (2004). Citrus fruit bitter flavors: Isolation and functional characterization of the gene *Cm1,2RhaT* encoding a 1,2 rhamnosyltransferase, a key enzyme in the biosynthesis of the bitter flavonoids of citrus. Plant J..

[42] Li G., Wen H., Zhou H., Liu Y., Yuan Z., Zhang H., Hu Z., Liu Z., Ma H., Chen Q. (2026). Evolutionary origin of neohesperidoside, a bitter metabolite, and its potential role in biotic defense and citrus dissemination. Plant Commun..

