# Supplementary material for: Metabolomics Reveals the Dynamic Characteristics of Flavonoids During the Different Developmental Stages of Citrus reticulata ‘Shiyue Ju’ and Its Mutant C. reticulata ‘Denglong Ju’
Source: Curr Issues Mol Biol. 2026 Jul 29;48(8):774. doi: 10.3390/cimb48080774 (PMC13511640; doi:10.3390/cimb48080774)
Supplement: Supplementary file 1 [file cimb-48-00774-s001.zip › Fig. S4.pdf]

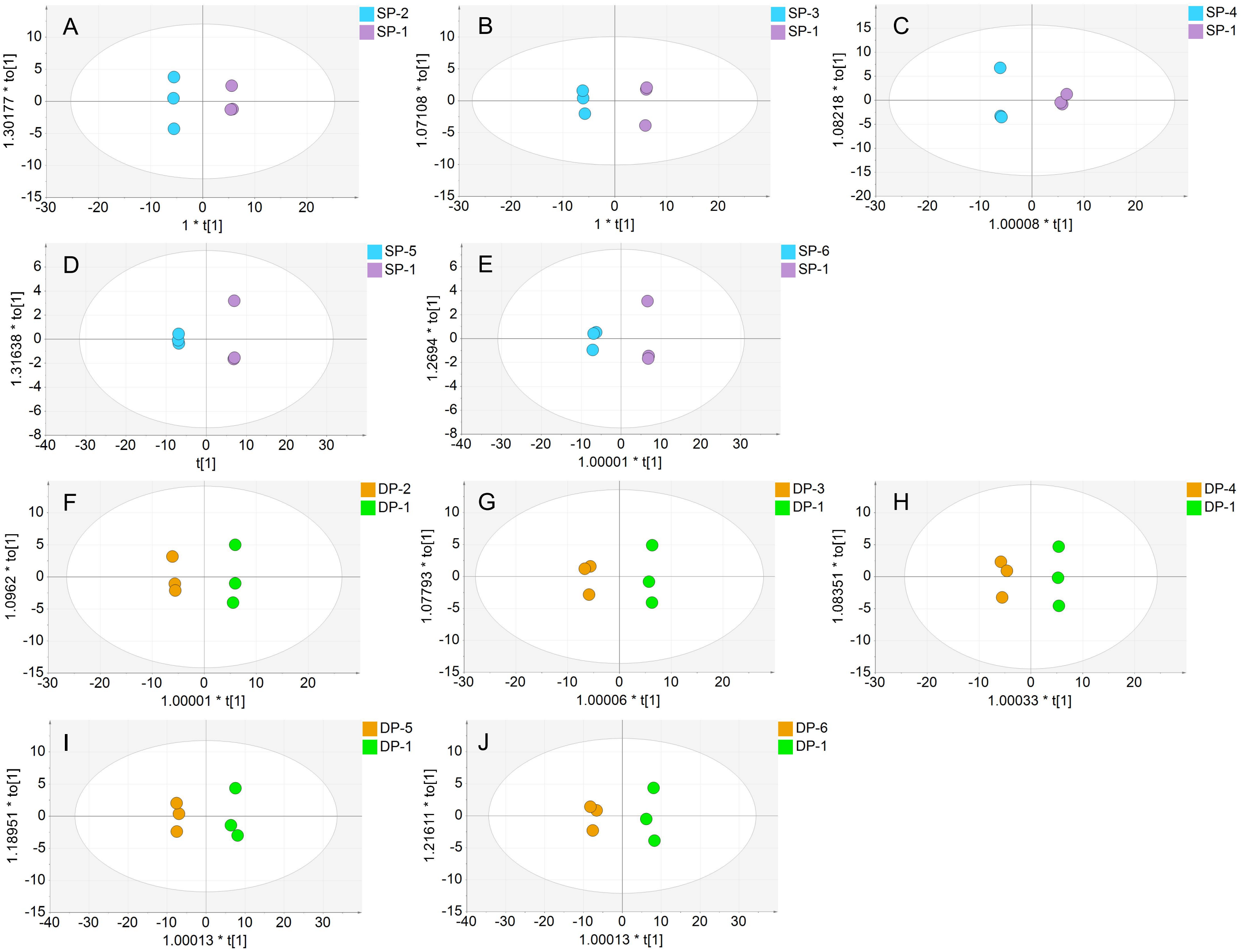

Fig. S4 OPLS-DA score plots in the all comparasion groups of SP and DP.
